# Protective effects of quercetin on liver injury induced by ethanol

**DOI:** 10.4103/0973-1296.62900

**Published:** 2010-05-05

**Authors:** Xi Chen

**Affiliations:** *Institute of Medicinal Plant Development, Chinese Academy of Medical Sciences, Peking Union Medical College, 151 Malianwa North Road, Haidian District, Beijing 100193, PR China*

**Keywords:** Quercetin, ethanol, liver injury, protective effect, rat

## Abstract

Quercetin, a natural compound of multiple origins, has broad biopharmacological effects, such as antioxidant, directly scavenging free radical, and hepatoprotectivity effects. This study is designed to investigate the interveneous effect of quercetin on liver injury induced by ethanol in rats. The rats that were orally treated with 50% ethanol for continuous ten days, which resulted in cell necrosis, fibrosis and inflammatory infiltration, were included in this study. Higher contents of AST, ALT ADH, γ-GT, TG in plasma and MDA in liver tissue, and lower content of GSH in liver tissue were highlighted in ethanol-treated rats when compared with healthy ones. The levels of cytokines such as IL-1β, IL-1, IL-6, IL-8, and TNF-α in rats plasma were also significantly enhanced, and level of IL-10 was obviously lowered through ethanol treatment. By preventive and synchronism treatment with quercetin for fourteen days, the contents of AST, ALT ADH, γ-GT, TG and MDA, and levels of IL-1β, IL-1, IL-6, IL-8, and TNF-α were significantly reduced, whereas GSH and level of IL-10 were obviously increased. It may be deduced that quercetin, by multiple mechanisms interplay, demonstrated somewhat protective effect on liver injury induced by ethanol in rats.

## INTRODUCTION

Alcoholic liver disease (ALD) remains one of the most common causes of chronic liver disease,[1] while ASD and chronic viral hepatitis are the leading causes of cirrhosis and hepatocellular carcinoma worldwide.[2,3] However, the mechanism of ethanol-induced liver injury associated with fatty liver, hepatitis, cirrhosis *etc* were not fully understood. Metallothionein is an intracellular protein, which is capable of binding metals and scavenging reactive oxygen species.[4,5] The main synthesis place of metallothionein is liver tissue,[6] and the studies revealed that ethanol is a potent inducer of liver metallothionein.[7,8] It is reported that several mediator systems are correlated with the development of ALD from fatty liver to advanced liver injury, such as inflammation[9] and necrosis. Alcoholic fatty liver is more susceptible to many inflammatory stimuli,[10] the bacterial endotoxins that are mainly involved in the inflammatory process. Therefore, in the experimental models of ethanol-induced liver injury, it has been demonstrated that endotoxin levels are correlated with liver pathology.[11,12] Moreover, oxidative stress or ischemic damage also seem to aggravate ethanol-induced hepatic inflammation.[13] Experimental evidences demonstrate that inflammatory reactions and oxidative stress play a major role in ethanol-induced liver injury.[14,15]

**Figure 1:**
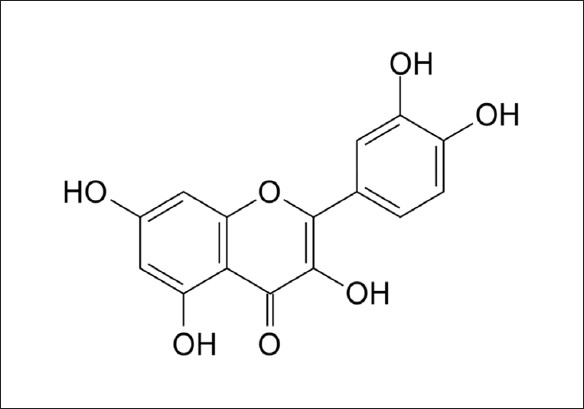
Chemical structure of quercetin

Although some important progresses have been made in investigating the pathogenesis of ALD, current treatments for this disease are not satisfactory. In recent years, it has been reported that the herbal drugs play a significant role in the therapy of hepatic disorders.[16–18] Quercetin, a flavonoid constituent[19] [Figure 1], is found in many herbal drugs and foods,[20] demonstrating broad biopharmacological properties.[21] Quercetin demonstrates antioxidant defense by scavenging free radicals and inhibiting various molecules oxidation,[22,23] being an useful agent for protecting various neuronal cells against oxidative stress.[24,25] Quercetin has preliminarily showed protective effect on liver injury in rats with carbon tetrachloride-induced cirrhosis,[26] but the study to investigate its antioxidant and hepatoprotective effects on ethanol-induced liver injury in animal model has been not carried out. The antioxidant defense of quercetin against oxidative stress action[24,25] is the main mechanism of its protective effect on ethanol-induced liver injury.[14,15] The purpose of this study is to assess the protective effect of quercetin on ethanol-induced acute liver injury in rats. Hepatoprotectivity can be achieved by eating foods which are rich in quercetin for reducing liver degeneration due to ethanol consumption

## MATERIALS AND METHODS

### Chemicals and reagents

Dehydrated ethanol was purchased from Beijing Chemical factory (Beijing, China). Quercetin (Purity>99%) was purchased from National Institute for the Control of Pharmaceutical and Biological Products (Beijing, PR China); the Assay Kits of glutamate-pyruvate transaminase (ALT), glutamic-oxal(o)acetic transaminase (AST) and triglyceride (TG) were purchased from Zhongsheng BeiKong Biological Technology Limited Corporation (Beijing, China); the Assay Kits of ethanol dehydrogenase (ADH) was purchased from Huili Biological Technology Limited Corporation (Changchun, China); the Assay Kits of nitric oxide(NO) γ-L- glutamyltranspeptidase (γ-GT, glutathion (GSH) and malondialdehyde (MDA) were purchased from Jiancheng Biological Engineering Institute (Nanjing, China); the Assay Kits of interleukin-1β (IL-1β), interleukin-1 (IL-1), interleukin-6 (IL-6), interleukin-8 (IL-8), interleukin-10 (IL-10) were from Schering-Plough Research Institute (Kenilworth, NJ). The Assay Kit of tumor necrosis factor α (TNF-α) was from BD Pharmingen (Los Angeles, CA). All other chemicals were of analytical grade.

### Animals and treatments

With the objective of finding out the protective effect of quercetin on acute liver injury in experimental animals induced by ethanol,[27,28] fifty male Wistar rats, weighing 240 ± 20 g, were purchased from the Animal Center of Shanhai SLAC Experimental and Animal Company (Shanghai, China). Animals were kept in an environmentally controlled breeding room (temperature: 24 ± 2°C, humidity: 60 ± 5%, 12 h dark-light cycles) for one week before the experiment. The rats were fed standard laboratory chow with water *ad. Libitum*. All animals were randomly divided into five groups including control group (CG), ethanol-treated group (EG), low dose group of quercetin (LG), middle dose group of quercetin(MG) and high dose group of quercetin (HG). The LG, MG and HG were treated with quercetin at a dose of 5 mg/kg, 10 mg/kg and 20 mg/kg by body weight, respectively, for continuous fourteen days. Fifty percent of ethanol (v/v) was administrated at a dose of 5g/kg after 2.5 h of quercetin administration from the fifth day for continuous ten days. An equal amount of distilled water was orally administrated for EG group for continuous four days, and 50% ethanol (v/v) at a dose of 5 g/kg from the fifth day for continuous ten days. The CG received an equal amount of distilled water for continuous fourteen days. On the fifteenth day, blood samples and liver tissues were collected from the rats under anesthesia for experimental analysis after 12 h of final treatment.

### Assay of cytokines and liver enzyme levels in rat serum

Blood samples were obtained from inferior caval vein, and serum IL-1β, IL-1, IL-6, IL-8, IL-10 and TNF-α amounts were measured by ELISA (R and D System, Abingdon, UK). Hepatocyte damage was evaluated by measuring serum enzyme activities of AST and ALT using an automated Synchron LX20 Beckman-Coulter according to the IFCC procedure[29,30] with the addition of pyridoxal phosphate.

### Assay of ADH, γ-GT and TG levels in rat plasma

All blood samples were collected from inferior caval vein of rats and centrifuged at 3000 rpm for 15 min, the supernatants were taken for ADH, γ-GT and TG assay. The activities of ADH, γ-GT and TG were measured by an automated Synchron LX20 Beckman-Coulter using diagnostic EIA kits according to the manufacturer's instructions.[31]

### Assay of MDA and GSH levels in rat liver

All liver tissues were perfused with 0.9% NaCl before cutting from rats for subtracting blood stain, and were homogenized on ice in 0.9% NaCl. The homogenates were centrifuged at 4500 rpm for 15 min at 4°C and the supernatants were taken for GSH and MDA assay.[32] The activities of GSH and MDA were measured by an Automated Synchron LX20 Beckman-Coulter using diagnostic EIA kits according to the manufacturer's instructions.[33,34]

### Statistical analysis

All data were expressed as means ±S.D. Significant differences among the groups were determined by one-way ANOVA analysis of variance using the SPSS 11.0 statistical analysis program (SPSS Institute, Cary, NC, USA). The *P* values<0.05 were considered as statistically significant.

## RESULTS

Effects of quercetin on cytokines in rats with ethanol-induced liver injury

**Figure 2:**
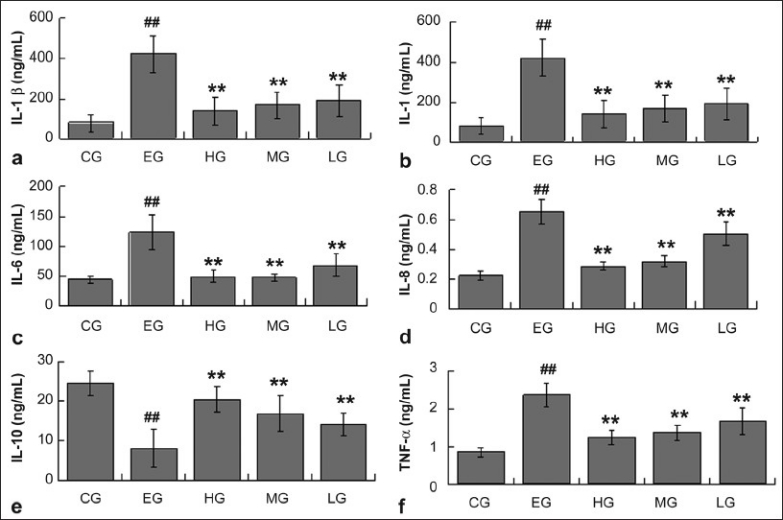
The levels of cytokines in serum from rats of CG, EG, HG, MG, and LG. Data are expressed as means ± SD for N = 10 rats/group. ##Significantly different from CG (*P* < 0.01) by two-way; **significantly different from EG (*P* < 0.01) by two-way; a, IL-β; b, IL-1; c, IL-6; d, IL-8; e, IL-10 f, TNF-α

In comparison with control group rats, the levels of IL-1β, IL-6, IL-8, and TNF-α were significantly elevated in rat serum administrated with ethanol, except IL-10 that showed an obvious decrease in serum level(P < 0.01). The quercetin-treated rats demonstrated the serum levels of IL-1β, IL-1, IL-6, IL-8, and TNF-α were obviously lowered, except IL-10 that showed an obvious increase compared with the ethanol administrated rats, and the effects of HG and MG were more remarkable [Figure 2]

### Effect of quercetin on levels of ALT, AST, ADH, γ-GT and TG in rats with ethanol-induced liver injury

**Figure 3:**
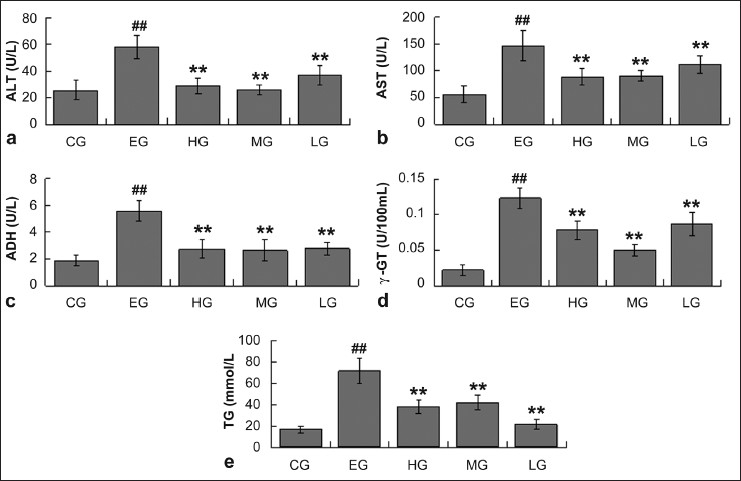
The levels of ALT, AST, ADH, γ-GT and TG in serum from rats of CG, EG, HG, MG, and LG. Data are expressed as means ± SD for N = 10 rats/group. ##Significantly different from CG (*P* < 0.01) by two-way; **significantly different from EG (*P* < 0.01) by two-way; a, ALT; b, AST; c, ADH; d, γ-GT; e, TG.

It is demonstrated that ethanol-treated rats produced severe liver injury by significantly increasing the serum levels of AST and ALT compared with that of the CG. All the rats treated with different doses of quercetin showed significantly decreased levels of ALT and AST compared with that of EG [Figure 3 a & b]. The serum levels of ADH, γ-GT and TG were also significantly elevated by ethanol administration. However, the rats treated with quercetin showed an obvious decrease in ADH, γ-GT and TG levels compared with that of EG [Figure 3 c, d & e].

### Effects of quercetin on levels of GSH and MDA in rats with ethanol-induced liver injury

**Figure 4:**
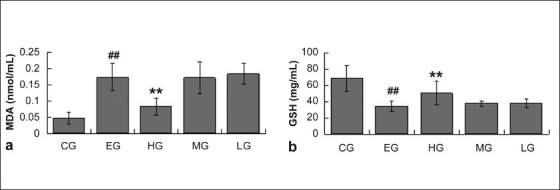
The levels of MDA and GSH in liver from rats of CG, EG, HG, MG, and LG. Data are expressed as means±SD for N = 10 rats/group. ##Significantly different from CG (*P* < 0.01) by two-way; **significantly different from EG (*P* < 0.01) by two-way; a, MDA; b, GSH.

The hepatic GSH level of rats administered with ethanol alone was found to be significantly lowered compared with that of CG, while rats treated with quercetin at a dose of 12 g/kg exhibited significantly increased hepatic GSH levels. The treatment groups of quercetin at a dose of 2g/kg or 6 g/kg almost did not prevent the decreases in hepatic GSH levels in comparison with that of EG [Figure 4]. A significant increase in hepatic MDA level was observed in ethanol-treated rats. However, ethanol-induced elevation in hepatic MDA level was lowered significantly when quercetin was administrated to the rats at a dose of 12g/kg, The MG and LG did not exhibit this effect [Figure 4] compared with EG.

### Effects of quercetin on levels of NO in rats with ethanol-induced liver injury

**Figure 5:**
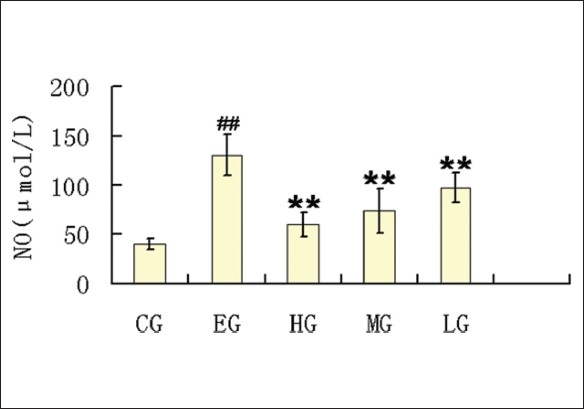
The levels of NO in serum from rats of CG, EG, HG, MG, and LG. Data are expressed as means±SD for N = 10 rats/group. ##Significantly different from CG (*P* < 0.01) by two-way; **significantly different from EG (*P* < 0.01) by two-way

It is reported that an acute high dose of ethanol induced an increase in NO levels.[35] It is found that ethanol treatment can significantly enhance the NO levels in rats plasma compared with that of CG. When different doses of quercetin were administrated, the levels of NO gradually decreased in CG rats. rats [Figure 5].

## DISCUSSION

Alcoholic liver disease (ALD) is associated with the overproduction of proinflammatory cytokines such as interleukin-1β(IL-1β), IL-1, IL-8 and IL-6.[36–41] These cytokines play a vital pathological role in the development of ALD, as demonstrated commonly in animal models, and mediates monocyte/Kupffer cell activation, elevated vascular permeability, necrosis and/or apoptosis of hepatocytes, over expression of adhesion molecules on endothelial cells, and the activation and chemoattraction of neutrophils and mononuclear cells.[42,43] From these studies, it can be found that ethanol treatment remarkably enhanced the levels of IL-1β, IL-1, IL-6 and IL-8 in rat serum; however, quercetin administration inhibited this elevations which is attributed to the significant reduction in the serum levels of IL-1β, IL-1, IL-6 and IL-8.

IL-10 is a potent anti-inflammatory cytokine that endogenously controls the synthesis of several proinflammatory mediators.[44] The levels of IL-10 in serum are obviously lowered with ethanol treatment, whereas quercetin administration has inhibited the reduction in the levels of IL-10, which is responsible for anti-inflammatory action and relieving liver injury in rats.

Ii is demonstrated that an acute high dose of ethanol consumption results in an elevation in NO levels,[35] the same increase in NO occurs in man who follow binge drinking.[45] The overproduction of NO is an important cause of inflammation reaction.[46,47] Moreover, the pathological role of TNF-α in ethanol- and endotoxin-caused liver injury, derived from experimental models, has also been demonstrated. On the one hand, chronic ethanol treatment in mice is correlated with elevatedexpression of TNF-α mRNA in the liver,[48] and mice with a targeted disruption of TNF receptor 1 are kept from ALD.[49] On the other hand, it is reported that, liver injury is essentially mediated by TNF-α in the GAL plus LPS model.[50] In this study, it is observed that levels of NO and TNF-α in rats serum were significantly enhanced by ethanol, whereas the increases were obviously inhibited by quercetin treatment, supporting the hepatoprotective effect of quercetin on ethanol-treated rats, which is partly attributed to its anti-inflammation action.

As we all know, liver Injury after ethanol treatment is a common phenomenon, and the obvious indicator of liver injury is the leakage of cellular enzymes into plasma.[51] The increase in serum enzymes levels associated with ALT and AST has been observed in ethanol-treated rats, which shows the enhanced permeability, injury and necrosis of hepatocytes.[52] The hepatoprotective effect of quercetin on liver injury is well evident, which significantly inhibits the increases in these enzymes levels caused by ethanol for keeping the liver structural integrity from ethanol injury.

Many researchers have demonstrated that ethanol-induced liver injuries are related to free radicals and oxidative stress.[53–55] Lipid peroxidation plays an important role in oxidative stress injury for liver,[56] which was determined indirectly by evaluating the enhances in MDA levels[57] and the decreases in GSH levels.[58] In this study, we observed a higher level of MDA and a lower level of GSH in the liver of ethanol-treated rats, which has been recognized as a proof to support the hypothesis that reactive oxygen intermediates, generated from the metabolism of ethanol, are attributed to lipid peroxidation and glutathione oxidation induced the ethanol hepatotoxicity. Quercetin-administrated rats showed significantly increased GSH level and decreased MDA level when compared with ethanol-treated rats, demonstrating the antioxidant effects of quercetin. We conclude that the hepatoprotective effect of quercetin may be partly due to its antioxidant activity.

Simultaneously, some studies demonstrated that the physiological factors that change liver ADH activity cause alteration in the rate of ethanol metabolism.[59–61] These evidences support the opinion that the level of ADH is a key factor controlling the metabolic rate of ethanol *in vivo*. The activity of γ-GT is an indicator of hepatic damage, which is usually used as sensitive marker in the diagnosis of hepatic diseases.[62] In this study, the serum levels of ADH and γ-GT were significantly enhanced by ethanol treatment, showing higher concentrations of ethanol in blood and certain injury in rat liver. In quercetin-treated rats, the serum levels of ADH and γ-GT were decreased significantly when compared with ethanol-treated rats. These results may support the fact that hepatoprotective effect of quercetin is partially attributed to its effect of accelerating ethanol metabolism and excretion.

In conclusion, the above analysis well supports the protective effect of quercetin on ethanol-induced acute liver injury in rats. We conclude that quercetin, by multiple mechanisms interplay, demonstrates hepatoprotective effect on liver-injury induced by alcohol, by increasing ethanol metabolizing enzyme activities, increasing antioxidant system activities against oxidative stress, lowering the expressions of proinflammation cytokines. This study also suggests the necessity of selecting some natural compounds for ALD therapy.
